# Supplementary material for: Endothelial Nucleoporin93 (Nup93) Maintains Vascular Function via Sun1-Dependent Regulation of RhoA-eNOS Signaling
Source: bioRxiv. 2025 Jul 21:2025.07.18.664980. Preprint. [Version 1] doi: 10.1101/2025.07.18.664980 (PMC12330544; doi:10.1101/2025.07.18.664980)
Supplement: Supplement 1 [file media-1.pdf]

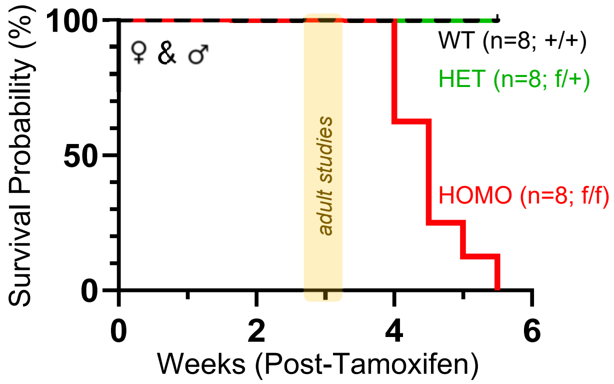

Figure S1. Endothelial loss of Nup93 is incompatible with life. Mice homozygous for the Nup93 floxed allele invariably face lethality irrespective of sex whereas heterozygous Nup93 floxed mice remain grossly unaffected. All *in vivo* adult studies were performed within 3 weeks of the last tamoxifen injection to avoid the morality time window (denoted in yellow).

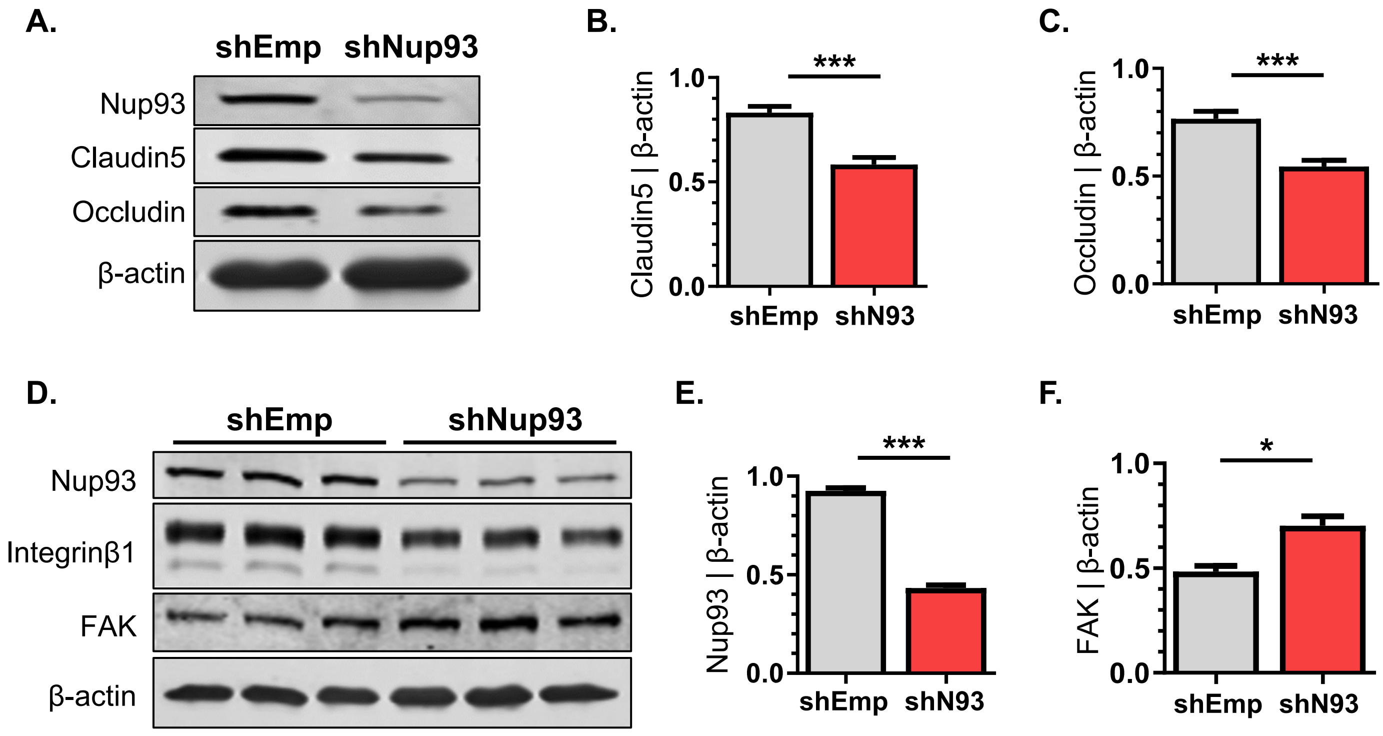

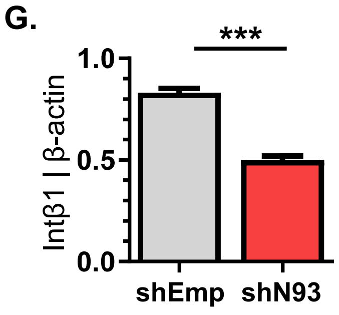

Figure S2. Endothelial Nup93 knockdown decreases tight junction protein expression and increases FAK levels. (A) Loss of Nup93 in primary ECs leads to decreased expression of tight junction proteins. Quantified in **(B)** and **(C)**. **(D)** Endothelial knockdown of Nup93 impacts integrin β1 and total FAK levels. Quantified in **(E-G)**. n=3, *** $p<0.001$, ** $p<0.01$, * $p<0.05$.

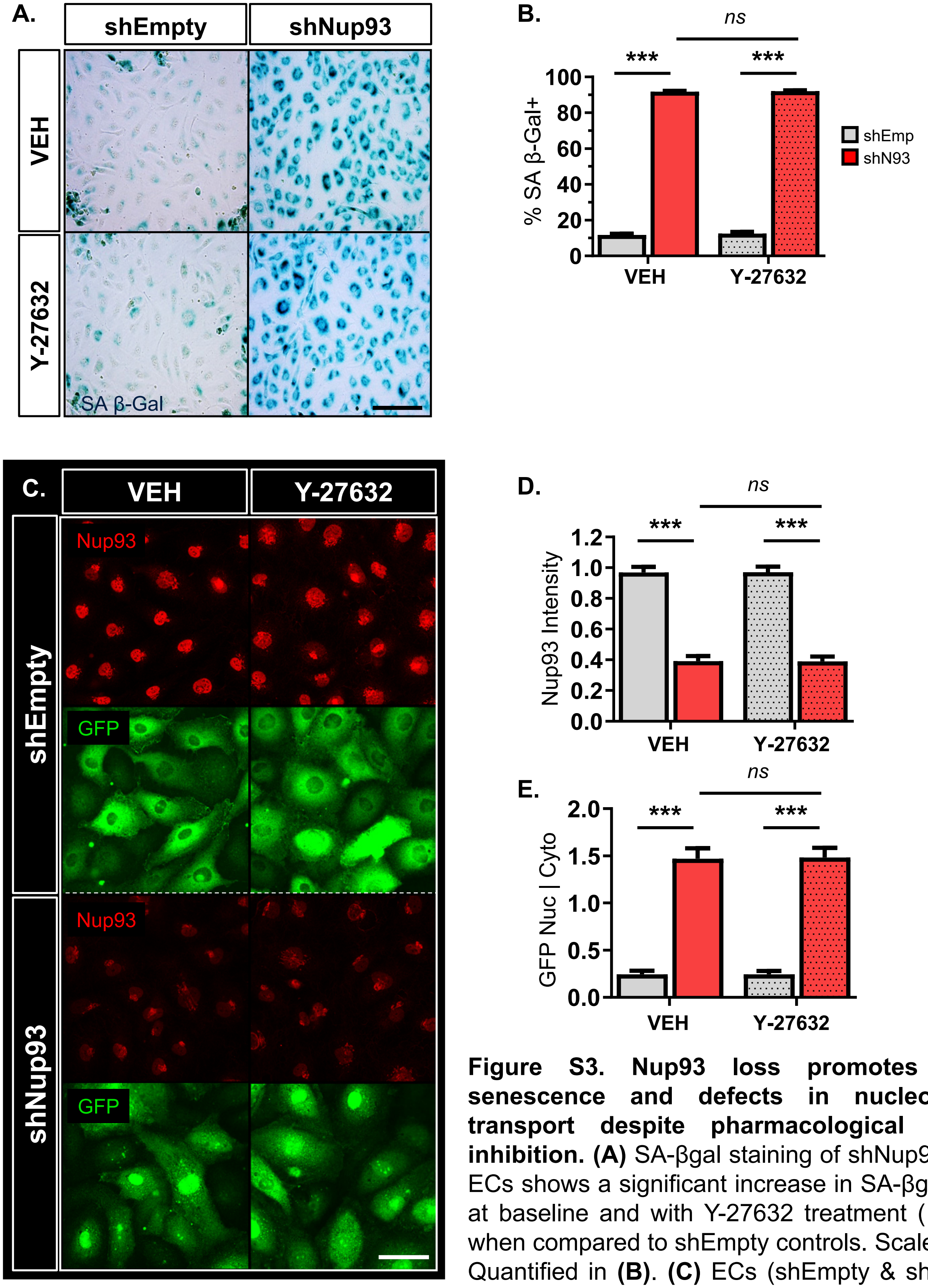

Figure S3. Nup93 loss promotes endothelial senescence and defects in nucleocytoplasmic transport despite pharmacological RhoA/ROCK inhibition. (A) SA-βgal staining of shNup93-transduced ECs shows a significant increase in SA-βgal signal both at baseline and with Y-27632 treatment (10μM; 24hrs) when compared to shEmpty controls. Scale bar=200μm. Quantified in **(B)**. **(C)** ECs (shEmpty & shNup93) were further transduced with the RGG construct using lentiviral methods followed by Y-27632 treatment (10μM, 24hrs). GFP nuclear-to-cytoplasmic intensity remains unaffected by Y-27632 treatment in Nup93 knockdown ECs when compared to vehicle-treated conditions. Scale bar=50μm. Nup93 expression and GFP nuclear-to-cytoplasmic levels quantified in **(D)** and **(E)**. n=3, *** *p<0.001*

Supp. Figure 4

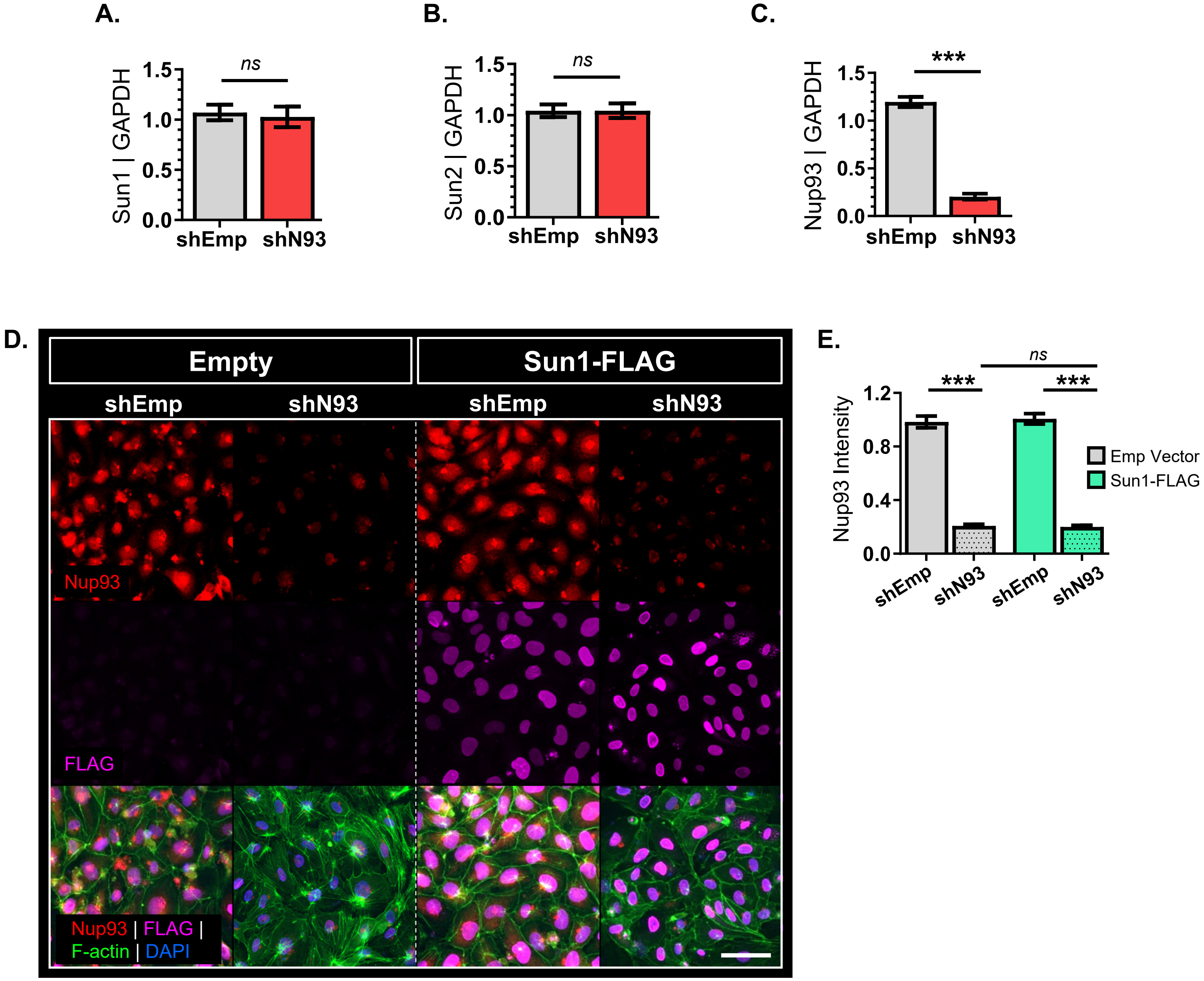

Figure S4. Targeted knockdown of endothelial Nup93 does not affect Sun1/2 transcript levels. (A-C) RT-qPCR analysis in primary ECs indicates a significant decrease in Nup93 mRNA levels when using shRNA-based method, whereas Sun1 and Sun2 expressions remain unaffected. **(D)** Immunofluorescence staining validates shRNA-mediated Nup93 loss and proper localization of exogenous Sun1 in both shEmpty control and shNup93 ECs, as demonstrated via nuclear FLAG signal. Nup93 signal quantified in **(E)**. Scale bar=50μm. n=3, *** *$p<0.001$*

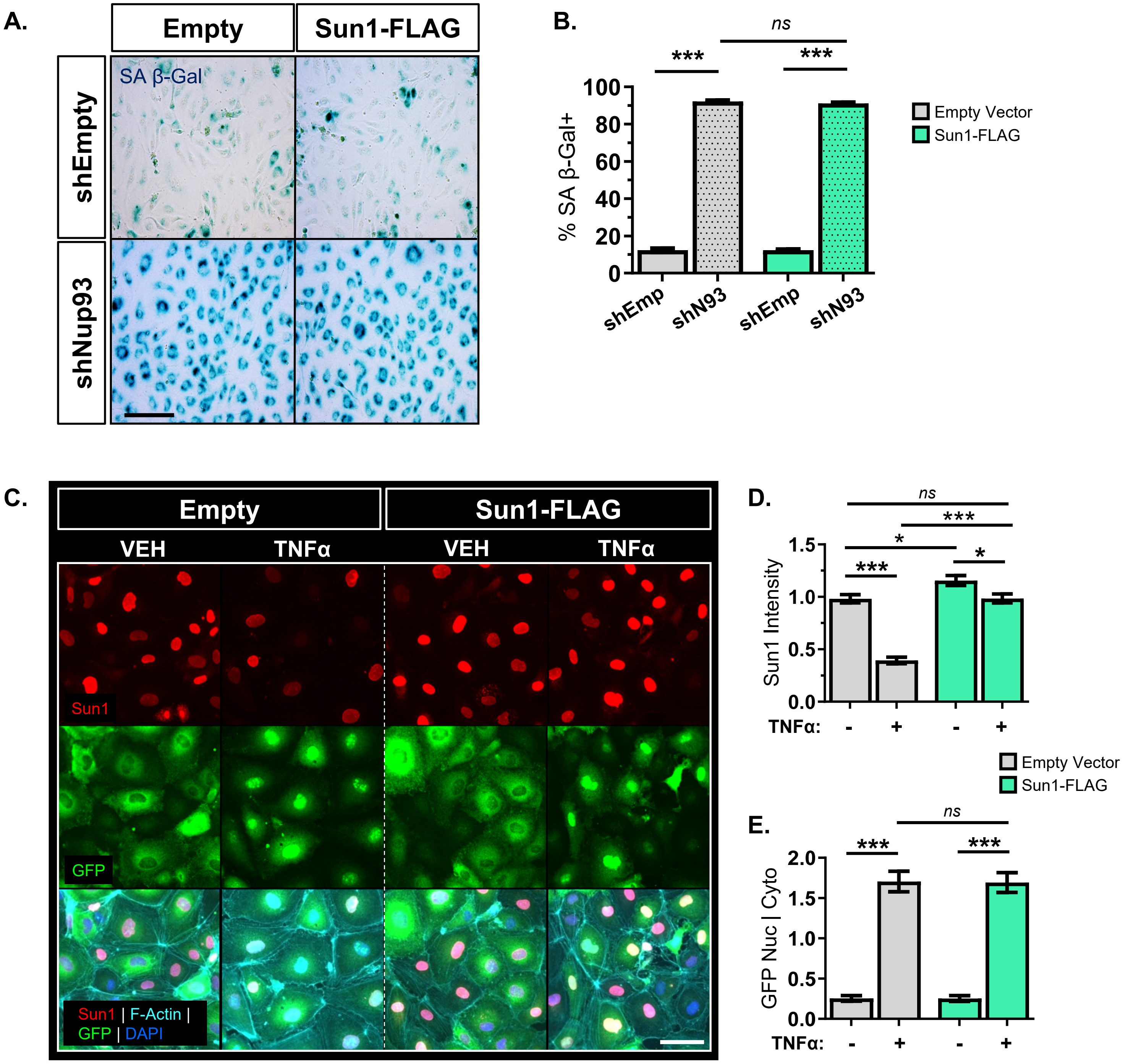

Figure S5. Nup93 loss triggers endothelial senescence and defects in nucleocytoplasmic transport despite restoration of Sun1 levels. (A) SA-βgal staining of shNup93-tranduced ECs shows a significant increase in SA-βgal signal regardless of exogenous Sun1 delivery. Scale bar=200μm. Quantified in **(B)**. **(C)** RGG-transduced primary ECs were first exposed to chronic inflammation (TNFα [10ng/mL]; 6 days) to induce EC senescence and nucleocytoplasmic transport defects. While long-term inflammation significantly reduces Sun1 expression, restoring Sun1 does not rescue NPC transport function; GFP nuclear-to-cytoplasmic intensity remains unaffected with exogenous Sun1 expression. Quantified in **(D)** and **(E).** Scale bar=50μm. n=3, *** *p<0.001, * p<0.05*

Table S1. Genotyping primers

Target Gene	Forward (5'-3')	Reverse (5'-3')	Product Size (bps)
Mouse			
Nup93 flox	TTTGGGTACCTTACTCCCACA	CAACCCAATGAGTCCCTTCT	552 (WT); 620 (flox)
Generic Cre *(JAX, oIMR1084 & oIMR1085)*	GCGGTCTGGCAGTAAAAACTATC	GTGAAACAGCATTGCTGTCACTT	100
Internal Control *(JAX, oIMR7338 & oIMR7339)*	CTAGGCCACAGAATTGAAAGATCT	GTAGGTGGAAATTCTAGCATCATCC	324

Table S2. RT-qPCR primers

Target Gene	Forward (5'-3')	Reverse (5'-3')	Product Size (bps)
Human			
NUP93	AGGACAATGCCCTGCTGTCT	AAGGGCGTCTTCTCCTGATG	152
SUN1	GGACGTGTTTAAACCCACGACTTCTCG	CTCTGACTTTAGCTGATCCAGCTCCAGC	457
SUN2	AAACTGCTGCTCGCATCC	GAGTCTTGCTGATGCTCTGCT	81
GAPDH	CTCTCTGCTCCTCCTGTTCGAC	TGAGCGATGTGGCTCGGCT	71
